# *Thalassophryne nattereri* fish venom: from the envenoming to the understanding of the immune system

**DOI:** 10.1186/1678-9199-20-35

**Published:** 2014-08-13

**Authors:** Monica Lopes-Ferreira, Lidiane Zito Grund, Carla Lima

**Affiliations:** 1 Unidade de Imunorregulação, Laboratório Especial de Toxinologia Aplicada (CEPID/FAPESP), Instituto Butantan, Av. Vital Brazil, 1500 São Paulo, SP, CEP 05503–900, Brasil

**Keywords:** Venomous fish, *Thalassophryne nattereri*, Immunological memory, Nattectin, Natterin

## Abstract

*Thalassophryne nattereri* (niquim) is a venomous fish found off North and Northeast coast of Brazil, where it is known by the severity of the accidents involving humans. This review article is divided into four topics. The first one provides a brief description of the animal biology and its distribution off Brazilian coastal waters, the venom apparatus, signs and symptoms observed in envenomated humans and also describes envenomation in mice. The second topic describes the use of modern genetic approach and mass spectrometry for identification of highly expressed genes in its venom glands and the sequence of major toxins. The third chapter offers a detailed study of tissue injury induced by the venom and reveals the role of toxins that impair inflammation reduction. Finally, the fourth section expands the understanding of many extrinsic and intrinsic essential factors in maintaining survival of memory B cell compartment. Our results demonstrate the wide possibilities for research in the area of toxinology, also the necessity of interconnection among biochemistry, pharmacology and immunology areas for the expansion of knowledge and for generation of innovation.

## Introduction

The Immunoregulation Unit has been developing research at the Butantan Institute since 1996 and began its work trying to clarify the pathophysiology of envenomation caused by the venomous fish *Thalassophryne nattereri*, common in the waters of the North and Northeast Brazilian coast. Initially, we reproduced in experimental animals the envenomation and could determine the pattern of injuries caused by the venom at histological and cellular levels. Similarly, we characterized the proteins that constitute the venom and determined their minimum lethal dose. Our efforts revealed that injuries caused by fish venoms differ from those induced by terrestrial venomous animals including snakes. *T. nattereri* fish venom provokes ischemic necrotic lesions of difficult regeneration that are poorly infiltrated by leukocytes such as neutrophils. There are changes in the structure of the extracellular matrix induced by activation of matrix metalloproteinases with decreased content of collagenous fibers during the healing phase of the lesion, which suggests the interference of the venom in mechanisms of recruitment and/or survival of inflammatory cells at the injury site.

The continuation of this line of research led us attribute these differences in the pattern of injuries to variances in venom composition. *T. nattereri* venom possesses a novel family of non-homologous and non-neurotoxic proteases – called natterins – that are unable to act on soluble components of the complement system, coagulation or even on platelets. Natterins are capable of cleaving the human kininogen and synthetic peptides derived from kininogen releasing Lys-BK or kallidin. Another particular component of the venom is nattectin, a C-type lectin unlike those found in snake venoms. It is a monomer that binds to galactose-terminated proteins and does not interfere with coagulation. Recent research has shown that envenomation symptoms are not reduced by the administration of anti-inflammatory drugs commonly used in clinical medicine (such as dexamethasone and indomethacin) neither by inhibitors of nitric oxide synthase (L-NAME) nor by serotonin antagonist (cyproheptadine), but partially only by administration of specific inhibitors of kallikrein and antivenom serum.

The results obtained from 1996 to 2000 by our group generated important knowledge on the pathophysiology of envenomation by *T. nattereri*, the biochemical nature of its toxins and therapeutic control of injuries. Our research also allowed a view of the complex network of interactions between the innate and specific immune system. Thus, it opened up a new perspective for future studies on this fish venom or its toxins as tools for intervention in the immune system. Our line of research aimed at better understanding the cellular and molecular immunological mechanisms underlying the inflammatory phenomena and their importance for the function of different tissues in murine experimental models. Using the venom of *T. nattereri* our group has succeeded in reproducing in mice the type of envenomation that occurs in humans and in establishing suitable models to study the cellular response of the innate compartment (inflammatory) and immune effector memory.

We evaluated whether natterins affect the leukocyte-endothelial cell interaction, hampering leukocyte mobilization and extravasation. The work started asking how the main toxins of *T. nattereri* venom contribute to the deficient influx of inflammatory leukocytes, which consequently leads to a delayed inflammatory reaction and healing of the injured tissue. It ended demonstrating that natterins may control the leukocyte-endothelial wall interactions in a mechanism that depends on negative signals derived from TLR2-TLR4/Myd88 signaling cascade. Interestingly, we confirmed that the antagonist effect of natterins is mediated by the activation of serine/threonine phosphatases and by the key signaling PI3K molecule. The unregulated toll-like receptors (TLR) signaling is associated with conditions such as septic shock, inflammatory diseases and cancer. A few studies have investigated how signaling events of cell surface (such as the binding of the CXC chemokine KC receivers) are influenced by the activation of TLR receptors, a likely event to occur during a microbial infection. Therefore, identification of mechanisms of action of endogenous or exogenous substances capable of inhibiting TLR-dependent signaling in macrophages, dendritic cells and endothelium is of great importance, and thus may define new molecular targets in acute and chronic inflammatory diseases.

In antigen-presenting cells such as dendritic cells, monocytes and macrophages, various inflammatory stimuli may trigger the phosphorylation of mitogen-activated protein (MAP) kinases. The fact that activation of these kinases could play an important role in regulating the effector function of these inflammatory cells, by influencing differentiation into activator or regulator subtypes of the immune response, led us to investigate the involvement of different signal transduction pathways dependent on MAPK induced by nattectin. We concluded that nattectin is a potential adjuvant candidate for activation of antigen-presenting cells due to its immunomodulatory ability to induce Th1-dominant responses.

Employing *T. nattereri* venom, we established an appropriate murine model for the study of cellular compartment of the immune memory response. Then, we used it to understand the hierarchical relationship between memory B cells and antibody-secreting cells (ASC) and for the understanding of the interrelationship of intrinsic factors, the microenvironment and different subtypes of memory T lymphocytes in controlling the memory response and the differentiation and maintenance of ASC.

Due to the high specificity of their targets, fish venoms and their toxins have been increasingly used as pharmacological tools in numerous experimental models and as prototypes for the development of new drugs. In 2006, in partnership with Cristália Pharmaceutical and Chemical Products [Cristália Produtos Químicos Farmacêuticos Ltda., Brazil] we made the deposit of a patent in Brazil, the TNP peptide with anti-inflammatory action, which has nowadays been deposited in European countries, USA and Asia. Preclinical trials have established the safety use of this peptide and its synthetic derivatives in the control of initiation and exacerbation of allergic asthma.

## Review

### *Thalassophryne nattereri* and the pathophysiology of envenomation

*Thalassophryne nattereri* (niquim) is a venomous fish of the Batrachoididae family. On the North and Northeast coast of Brazil it is known by the severity of the accidents involving fishermen and bathers, estimated as hundreds per year [1-3]. However, registered cases do not represent the real number of accidents caused by this fish in different regions of Brazil.

*T. nattereri* is small (12–15 cm), with large protruding eyes, large head and wide mouth. It does not have scales and the whole body is covered by a thick sticky mucus. This fish is predominantly found in marine and river brackish waters, living among rocks or seaweed and covered by sand or mud in a relatively shallow place. It has the habit of living buried in sand, but may be noticed by the outline of its body. Since it is carnivorous, it consumes mostly crabs, mollusks and small fish. It typically lives in groups and is extremely resistant, remaining alive out of the water for up to 12 hours. Its body color may change, by mimicry, to hide it from predators or prey.

**Figure 1:**
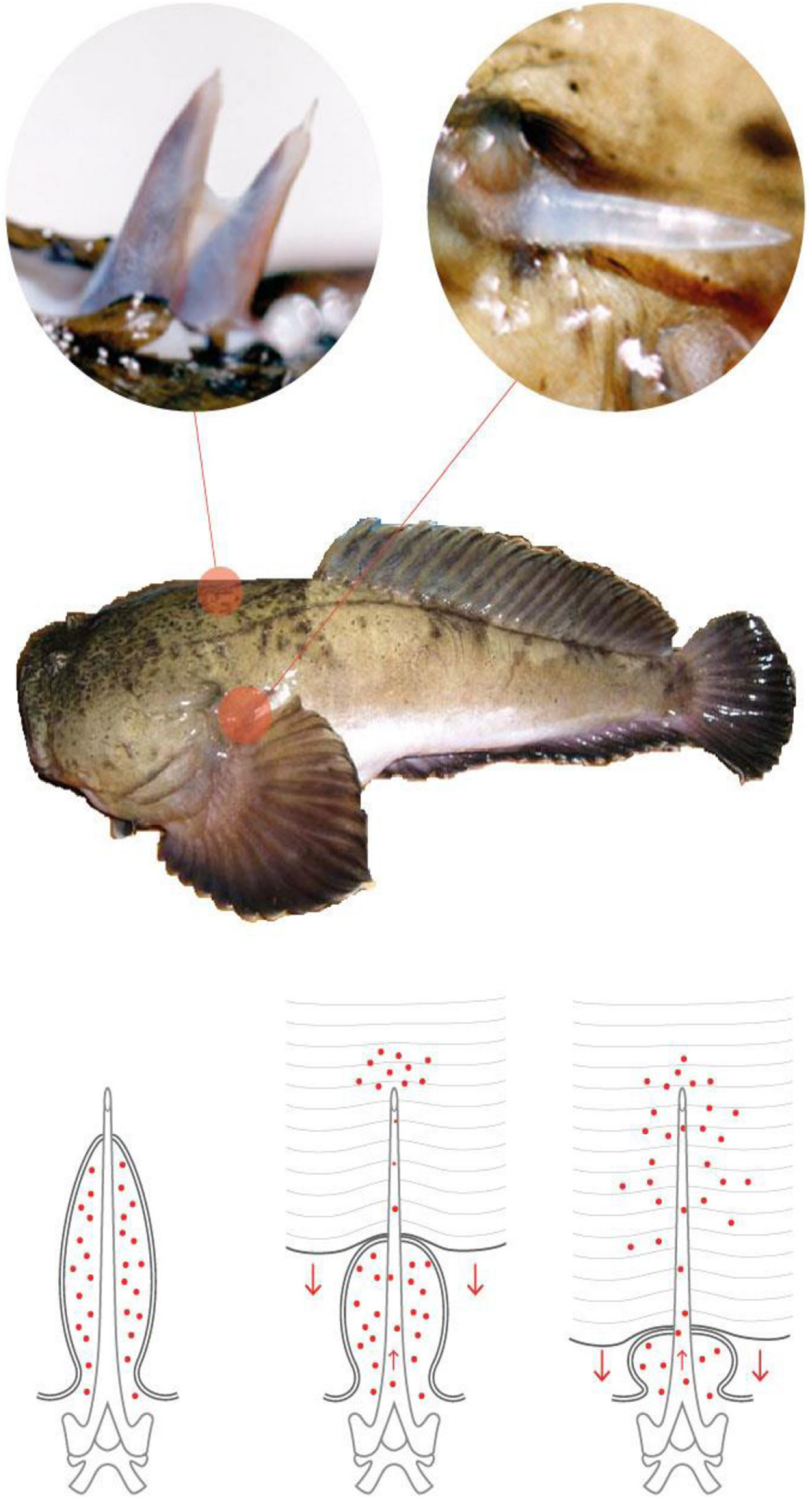
**The venom apparatus of *Thalassophryne nattereri *is composed of two dorsal and two lateral canaliculated spines covered by a membrane connected to the venom glands at the base of the fins.** When the spine penetrates the victim, the integumentary sheath enclosing the gland press out the venom into the duct.

**Figure 2:**
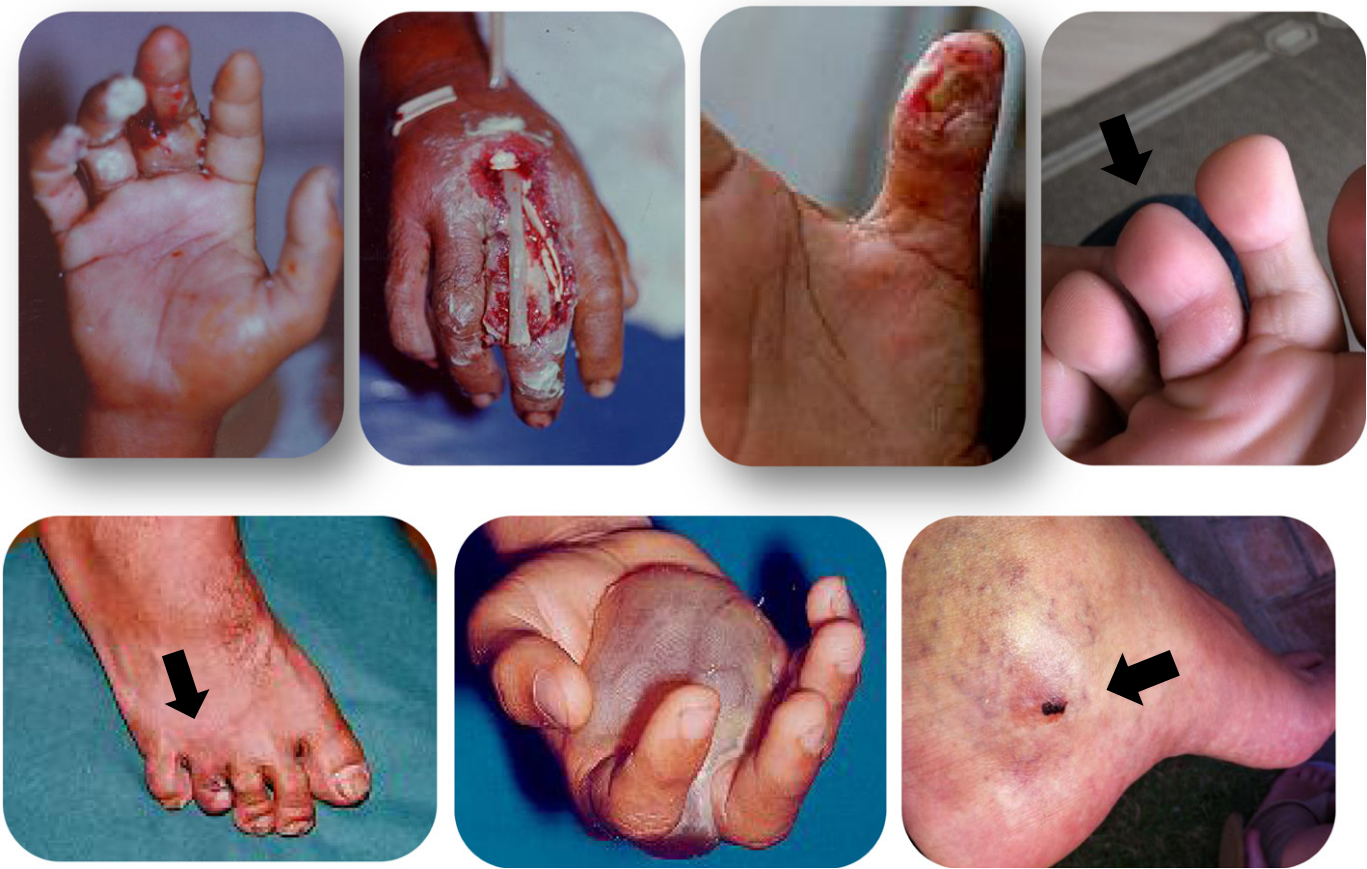
**The main manifestations of *T. nattereri *envenomation.** Hands and feet are the most commonly affected areas in humans, which usually present intense necrosis and markedly poor healing response.

Its venom apparatus is composed of two dorsal and two lateral canaliculated spines covered by a membrane connected to venom glands at the base of the fins (Figure 1). When the spine penetrates the tissue of victims, the integumentary sheath enclosing the gland press out the venom into a duct and the venom is injected into the victim [4]. According to Fonseca and Lopes-Ferreira [1], the palm of the hands and the soles of the feet are the most commonly areas affected in humans. The main symptoms of *T. nattereri* envenomation include local edema and rapidly developing excruciating pain, followed by intense necrosis and a markedly poor healing response. The inefficient healing is very significant for the evolution of the injury, which is devoid of specific treatment and drug therapies (Figure 2). Most accidents with *T. nattereri* venom occur in fishing communities and, due to the lack of efficient therapy, victims may take weeks, or even months before returning to work.

More than simply describing the species, its habitat and venom apparatus, Fróes [5] also reproduced the clinical symptoms of *T. nattereri* envenomation in guinea pigs and birds. Thereafter, we started in 1996, at the Butantan Institute, an intensive investigation for biochemical characterization and molecular identification of venom toxins and for evaluation of the inflammatory response present in sting sites.

We initially observed that intradermal injections of low doses of venom (0.3 μg/footpad) into mice induced local effects as pain, edema, and necrosis similar to those described in humans. Subcutaneous injections of the venom induced systemic effects consisting of jerking movements, paralysis of hind limbs, erection of hair, rotational movements and violent convulsions followed by death. Dead animals showed hyperemia of the small intestine and lungs.

The venom also promoted hemolytic activity, low level of hemorrhage, myotoxic and proteolytic activities and was devoid of phospholipase A2 activity [6]. *T. nattereri* venom induced direct damage on the skeletal muscle plasma membrane together with thrombosis and other alterations in the microvasculature of mouse cremaster muscle [7]. Afterwards, we expanded the study on the necrotic process induced by the venom introducing morphological and biochemical aspects of muscle damage and regeneration after intramuscular injection with *T. nattereri* venom in mice. Several muscle cells presented a hypercontracted morphology, but most necrotic fibers were not hypercontracted, being instead characterized by a disorganization of myofibrils, with Z-line loss, mitochondrial swelling and sarcolemmal disruption.

In addition, thrombosis was observed histologically in venules and veins, together with vascular congestion and stasis, evidenced by intravital microscopy. Venom induced a rapid increment in serum creatine kinase (CK) levels, concomitant with a reduction in gastrocnemius muscle CK activity, whereas no increments in muscle lactic acid were detected. A rapid cytolytic effect was induced by the venom on C_2_C_12_ murine myoblasts in culture.

**Figure 3:**
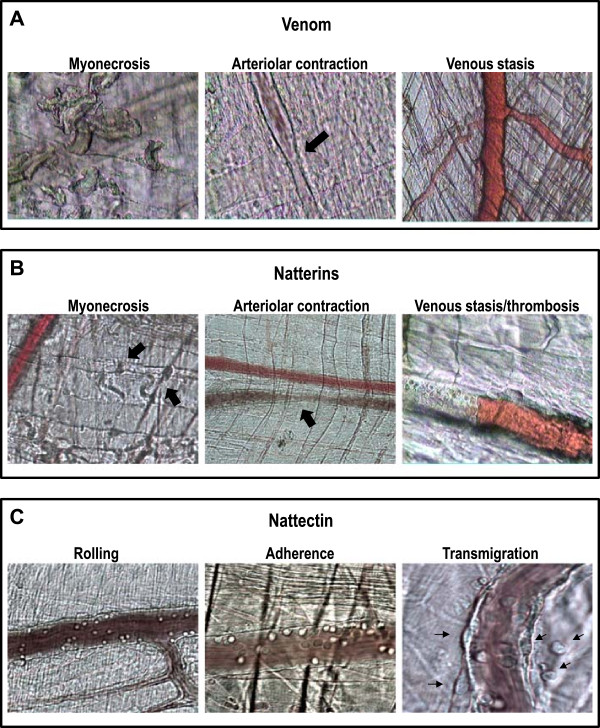
**Microcirculation of the cremaster muscle. Intravital microscopy analyses of the microvascular network of cremaster muscle submitted to intraescrotal injection of (A) venom, (B) natterins and (C) nattectin.** Venom and toxins elicits a peculiar alteration of the microcirculation that includes effects on platelets, endothelial cells and leukocytes.

The inflammatory reaction of affected muscles was characterized by scarce cellular infiltrate of polymorphonuclear leukocytes and macrophages, with a consequent delay in the removal of necrotic material. Skeletal muscle regeneration was partially impaired, as evidenced by the presence of regenerating fibers of variable sizes and by the increase of fibrotic tissue in endomysium and perimysium. These data suggest that *T. nattereri* venom affects muscle fibers by a direct cytotoxic effect, and that the vascular alterations described preclude a successful regenerative process [7] (Figure 3A).

Intravital microscopy analyses of the microvascular network of cremaster muscle showed effects on platelets and endothelial cells. Stasis was observed concomitantly with the presence of thrombi in venules and focal transient constrictions in arterioles, all of which impaired the blood flow. Significant alterations on vessel walls took place few minutes after venom administration, with thickness increase and deposition of fibrin. Augmentation of vascular permeability was also observed in venules. No alterations on systemic blood coagulation was noticed. The venom lacked a direct procoagulant activity, but exerted a strong cytolytic effect on platelets and endothelial cells *in vitro*[8]. These results suggest that venom action on endothelium may contribute to blood stasis and to the formation of platelet and fibrin thrombi, with consequent ischemia, contributing to the local injury and delayed regeneration (Figure 3A).

Anti-inflammatory drugs are inefficient in controlling symptoms of envenomation by *T. nattereri*, and most patients are treated with empirical procedures. Therefore, many cases evolve to permanent sequelae. A better understanding of the mechanisms that regulate the acute inflammatory events could lead us to the development of efficient therapeutic strategies. By means of experimental models that reproduce human accidents, we showed that venom-induced nociception and edema were not reduced neither by treatment with inhibitors such as indomethacin (a cyclooxygenase inhibitor), dexamethasone (a steroid anti-inflammatory agent), cyproheptadine (antagonist of serotonin receptors) nor L-NAME (inhibitor of nitric oxide synthase).

Both nociceptive and edematogenic responses were reduced after treatment with a specific tissue kallikrein inhibitor (TKI) by 78% and 24% respectively when compared to control values. Administration of a specific plasma kallikrein inhibitor (PKSI527) did not affect the venom-induced nociceptive response, but it decreased paw edema by 15%. The peptidase activity of *T. nattereri* venom was determined using internally quenched fluorogenic substrates containing sequences derived from human kininogen or peptides containing EDDnp, and the results showed the release of Lys-bradykinin. This suggests that general homeostatic mechanism rather than conventional inflammatory pathways are involved in the local effects of the venom [9].

Additionally we demonstrated that *T. nattereri* venom (0.3, 1.0, and 3.0 μg/mL) promoted increase in perfusion pressure and renal vascular resistance in mice with hyalin casts inside all tubules and proteinaceous material in the urinary space. We may suggest that the venom affects directly kidney cells by causing the release of vasoactive factors and the decrease of electrolyte transport [10,11].

### Transcriptome and proteomic analysis of venom and toxins

Aiming at better understanding the nature of *T. nattereri* venom toxicity, we carried out a structural characterization of the major venom toxins through proteomic and transcriptomic techniques. First, a novel family of proteins with kininogenase activity, the natterins, with unique primary structure was characterized using combined pharmacological, proteomic and transcriptomic approaches. The major venom components were isolated and submitted to bioassays corresponding to its main effects: nociception, edema, myonecrosis, arteriolar contraction and venular stasis (Figure 3B).

We describe a transcriptome analysis of 775 expressed sequence tags (ESTs) which represents a general panorama of the transcripts responsible for the physiological events that take place in the venom gland, since those data were generated from a non-normalized cDNA library. Although there are many sequences from other fish in databases – such as *Takifugu rubripes*, *D. rerio*, *S. salar*, *Oryzias latipes* and *Gasterosteus aculeatus* – this is the first description of a fish venom gland transcriptome, which will help to support comparative studies for other fish venom glands.

**Figure 4:**
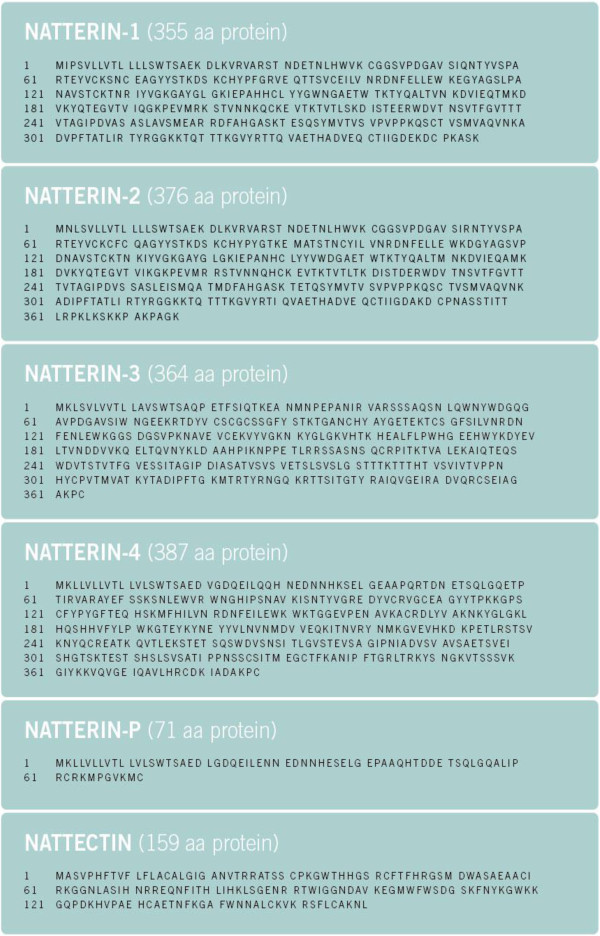
**Primary structure of toxins was obtained by a transcriptomic approach using a representative cDNA library constructed from *T. nattereri *venom glands.** This approach also allowed us to prove the existence of natterins (1–4 and P), a novel family of kininogenases, and a toxic C-type lectin nattectin.

The primary structure of natterins was obtained by a transcriptomic approach using a representative cDNA library constructed from *T. nattereri* venom glands. First, several ESTs were obtained and processed by bioinformatics tools and revealed a major group (18%) of related sequences unknown in gene or protein sequence databases. This group that included sequences showing the N-terminus of isolated natterins was called the natterin family. Analysis of this family allowed us to identify five related sequences, which we named 1–4 natterin and P-natterin. Natterin 1 and 2 sequences include the N-terminus of the isolated natterins (Figure 4). Furthermore, internal peptides of natterins 1 to 3 were found in major spots of whole venom submitted to mass spectrometry/2DGE. Similarly to ESTs, the complete sequences of natterins did not show any significant similarity with already described tissue kallikreins, kininogenases or any proteinase, all being entirely new [12,13].

Another cluster called nattectin (Figure 4), containing six ESTs, showed significant similarity with C-type lectins from other sources, particularly with C-type lectin from non-venomous fish such as *Spirinchus lanceolatus* and *Salmo salar* with identities around 34%, and with C-type lectins found in snake venoms from *Trimeresurus stejnegeri* and *Lachesis muta* with identities around 30%. Nattectin was also isolated from the venom. It is a basic non-glycosilated 15-kDa monomeric protein. It exhibits hemagglutination activity that is independent of Ca(2+) with remarkable proinflammatory activity, inducing neutrophil mobilization in mice [14] (Figure 3C).

### Innate response induced by venom and toxins and negative control of the inflammation

Previous work on the biochemical characterization and molecular identification of venom toxins, and evaluation of the inflammatory response in tissue injured by *T. nattereri* venom led to the construction of a methodological tool. It was useful for a better understanding of the importance of the innate cellular response in the control of toxins and consequently in restoration of the integrity of damaged tissue.

The injection of venom into footpad of mice increases the mRNA for IL-1, IL-6 and TNF-α genes as well as the release of these soluble cytokines. Moreover, absence of leukocytes infiltration in the intraplantar region of footpad of mice after venom application was also reported [15]. Moreover, Pareja-Santos *et al.*[16] showed that *T. nattereri* venom alters the extracellular matrix structure of mouse footpad tissue by the activation of matrix metalloproteinases 2 and 9 (MMP-2 and MMP-9), and it decreases collagen fiber production during the healing phase. It was also shown that the venom affects the cytoskeleton organization and pseudopodia formation of epithelial cells in *in vitro* system.

Recently, Komegae *et al.*[17] demonstrated the specific ability of nattectin to bind types I and V collagens and natterins to bind and cleave type I collagen as well as type IV collagen, disrupting cell attachment and HeLa cells survival. Natterins have cytotoxic effect on both adherent cells or on those in suspension, showing direct induction of necrosis that is followed by cell detachment. Nattectin improves integrin-mediated HeLa cell adhesion and resistance to apoptosis by its binding to RGD-dependent integrins, especially the β1 subunit. Based on our studies, we reported that extracellular matrix (ECM) components as well as the integrin β1 subunit are targets for natterins and nattectin. The ECM degradation or remodeling activities exerted by these toxins affect cell-cell and cell-ECM adhesion and survival and impair inflammatory cell migration into inflamed tissues.

This scenario indicates an ambiguous role of the venom in the inflammatory process. On one hand, it displays a potent proinflammatory activity illustrated by the detected chemoattractants up-regulation, and on the other hand, it affects the ability of tissue healing due to the extracellular matrix disorganization caused by MMPs up-regulated activity and by defective infiltration of inflammatory cells. Nevertheless, the reasons why venom favors delayed local inflammatory response or why a particular group of venom toxins directly controls the traffic of leukocytes into inflamed tissue are still under investigation.

Signals from the innate response may influence the physiology of macrophages, and these changes allow them to participate in homeostatic processes such as tissue remodeling and wound healing as well as in adaptive responses. Cytokines, growth factors and endogenous danger signals have been implicated in the reprogramming of M1 and M2 macrophages in tissue. As reported by Lima *et al.*[15] an influx of mononuclear cells into footpad of mice was observed as a delayed response and at that time the levels of IL-6, IL-1β and MMP-2 remained high. Then, we investigated the modulatory capacity of nattectin in this important innate cell type.

We have shown that the phenotypic changes in peritoneal and bone marrow-derived macrophages induced by nattectin are consistent with the classical M1 activation (iNOS expression), dependent on Th1 cytokines (IL-12 and IFN-γ), and negatively regulated by Th2 cytokines (IL-4, IL-10 and IL-13). The increase in MHC class II expression with augmented expression of CD49a integrin, MMP-9 production and endocytic capacity depend on the lectin function of nattectin, while its effects on co-stimulatory molecules up-regulation are partially PI3K and p38 MAPK mediated. The endocytic capacity of macrophages induced by nattectin is promoted by its binding to glycosylated structures expressed in these cells and also depends on ERK1/2 signaling pathway. Our results show that nattectin provokes an increase in the expression of α1 and α5 integrins, CD44 and MMP-9, which supports the idea that nattectin-activated macrophages acquire a competent molecular complex for migration that qualifies them to access tissue [18].

Saraiva *et al.*[19] confirmed the potent proinflammatory properties of nattectin when they demonstrated that macrophages recruited to the peritoneal cavity by nattectin differentiated into mature dendritic cells (DC), as evidenced by up-regulation of Ly6C, F4/80R, CD80 and MHC class II. These macrophage-derived DCs exhibit functional attributes (augmented antigen presentation capacity with avid phagocytosis, high T cell co-stimulatory molecules expression, and enhanced capacity to activate T cells by the production of IFN-γ through the high-level of bioactive IL-12 p70 secretion) that are associated with Th1 differentiation. Our *in vivo* results confirmed that nattectin induced a Th1 polarized response, characterized by high levels of specific IgG2a and antigen-specific IFN-γ-producing cells.

More recently, we evaluated whether natterins affect the leukocyte-endothelial cell interaction, hampering leukocyte mobilization and extravasation. Leukocyte-endothelial cell interactions were evaluated in venules of mouse cremaster muscle using intravital microscopy. We observed that low doses of natterins affect cell capturing and inhibit the interaction of blood neutrophils with post-capillary venules induced by the TLR4 agonist lipopolysaccharide (LPS), or the chemokine KC. Using endotoxemic mice challenged with LPS, we confirmed that natterins reduce neutrophil accumulation in the peritoneum exudates. The rolling of leukocytes induced by KC or LPS was not impaired in natterins-treated TLR2, MyD88 deficient or TLR4 mutant mice, indicating that TLR2- or TLR4-MyD88-mediated signals are required for the anti-inflammatory effect of natterins. The inhibitory effect was not influenced by endogenous regulators of inflammation such as IL-10, corticosteroids, HO-1 neither by the antagonist of the receptor of IL-1, nor by the disruption of their proteolytic activity. However, it was completely dependent on the activation of serine/threonine phosphatases and the PI3K signaling pathway, but independent of increased proteasome activity [20].

These works started asking how the main toxins of the *T. nattereri* venom contribute to the deficient influx of inflammatory leukocytes, which consequently leads to the delayed inflammatory response in injured tissue. By their end, we demonstrated that natterins may control the leukocyte-endothelial wall interactions in a mechanism dependent on negative signals derived from TLR2-TLR4/Myd88 signaling cascade. Thus, our data allowed us to expand the knowledge on the regulation of cell activation and transmigration, as well as confirmed that nattectin and natterins comprise important immunomodulatory agents [7,8,14-20].

### Humoral response and B cell compartment

Similarly to therapeutic approaches employed against envenomation provoked by fish in Indic oceans [21-23], we investigated the potential use of antiserum against *T. nattereri* venom as suggested by Auto [24]. Lopes-Ferreira *et al.*[25] gathered evidences that support the efficacy of *T. nattereri* antiserum produced in rabbits. The antiserum was completely able to inhibit nociception and necrosis when administered minutes after the venom injection. Subsequently, we confirmed that mice immunized twice with *T. nattereri* venom with high levels of circulating IgG have refractory capacity to develop nociception, necrosis and edema induced by subsequent venom injection in the footpad. The mice developed less thrombi in venules and vascular constriction in arterioles [26].

These findings allowed us to explore specific antivenom as a therapeutic strategy for human victims. Subsequently, we produced antiserum in horses and evaluated the effectiveness of its different isotypes in the neutralization of the main toxic activities induced by venom [27]. Preincubation of venom with whole antiserum or isolated IgG had the most potent capacity to neutralize lethality, nociception, necrosis and microcirculatory alterations induced by venom. However, it was only partially capable of neutralizing edema. The murine model of humoral response induced by venom allowed the study of memory B cell compartment.

**Figure 5:**
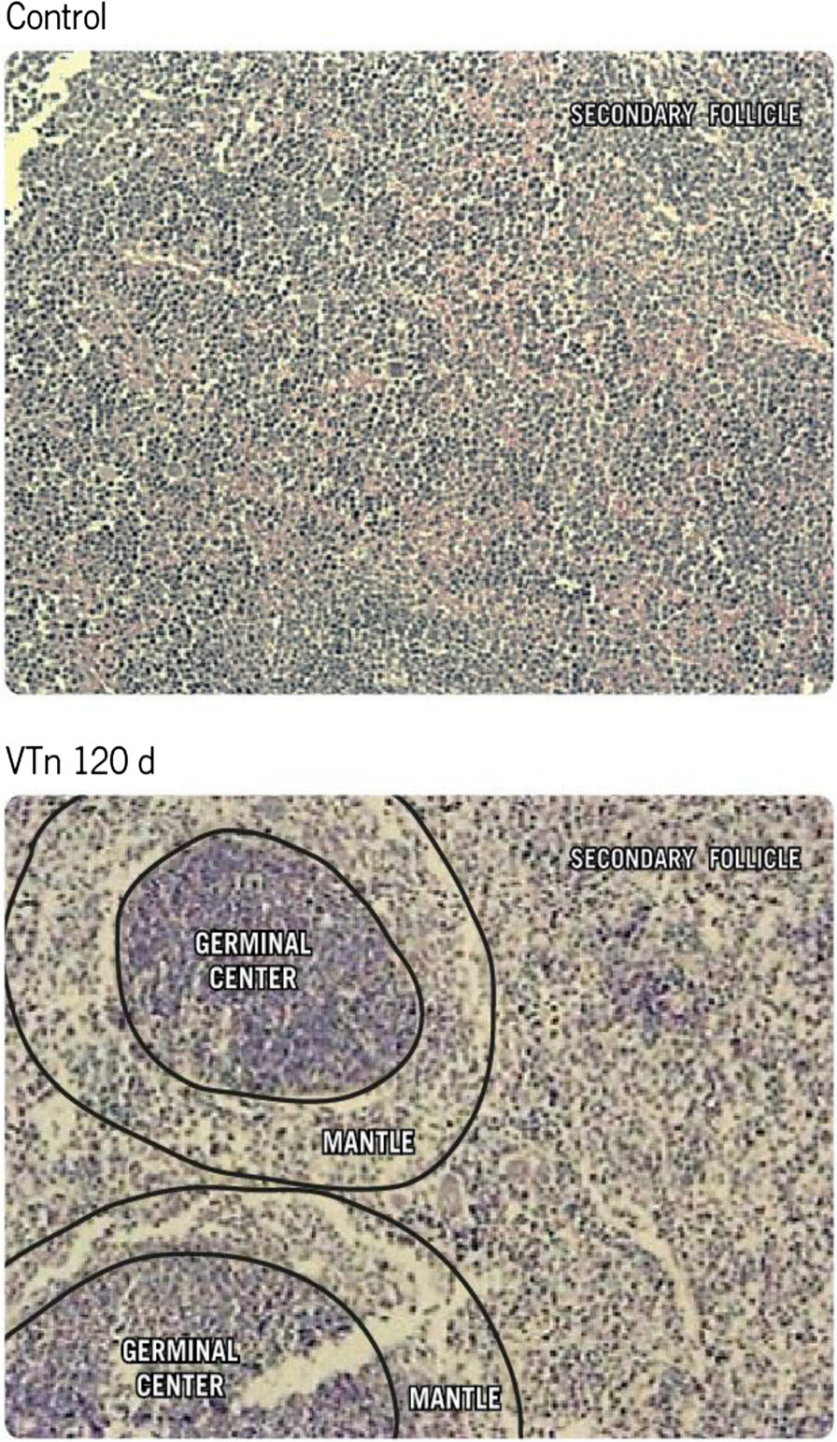
**Venom promotes germinal center formation.** BALB/c mice were immunized i.p. with 10 μg of *T. nattereri* venom adsorbed in Al(OH)_3_ on day 0 and boosted on day 14 with the same dose of venom. Animals injected only with Al(OH)_3_ were considered as control group. After 120 days of immunization is observed typical B cell areas in lymphoid follicles of spleen showing an increased expansion of germinal centers compared with than control-mice.

Grund *et al.*[28] demonstrated in mice that the venom elicited high levels of venom-specific IgG1 and total IgE and low levels of specific IgG2a, accompanied by IL-5 and IFN-γ production by splenic lymphocytes. These data confirmed that *T. nattereri* venom stimulates both Th1 and Th2 immune response and has a substantial influence on the magnitude and quality of such responses. Venom protein content is predominantly composed of natterins and nattectin, as demonstrated by Magalhães *et al.*[13], and each one of them may elicit Th1 or Th2 response. The high levels of detected IL-5 indicate that venom is a strong stimulus for generation of memory B cells and ASC from committed B-2 B cells in germinal center in secondary lymph organs (Figure 5).

The precise mechanisms by which innate germinal center (GC) differentiation, affinity maturation and longevity of the humoral or cellular responses are modulated are still poorly understood, but several studies have identified critical roles for innate molecules and cells in this process. From a combined *in vivo* and *in vitro* approaches, we have demonstrated for the first time that IL-5 and mainly IL-17A – produced in a situation of chronic inflammatory response against venom proteins – directly influence the production of specific IgE antibodies and the maintenance of ASC with B220^neg^ phenotype outside the germinal center, in inflamed peritoneal cavity.

**Figure 6:**
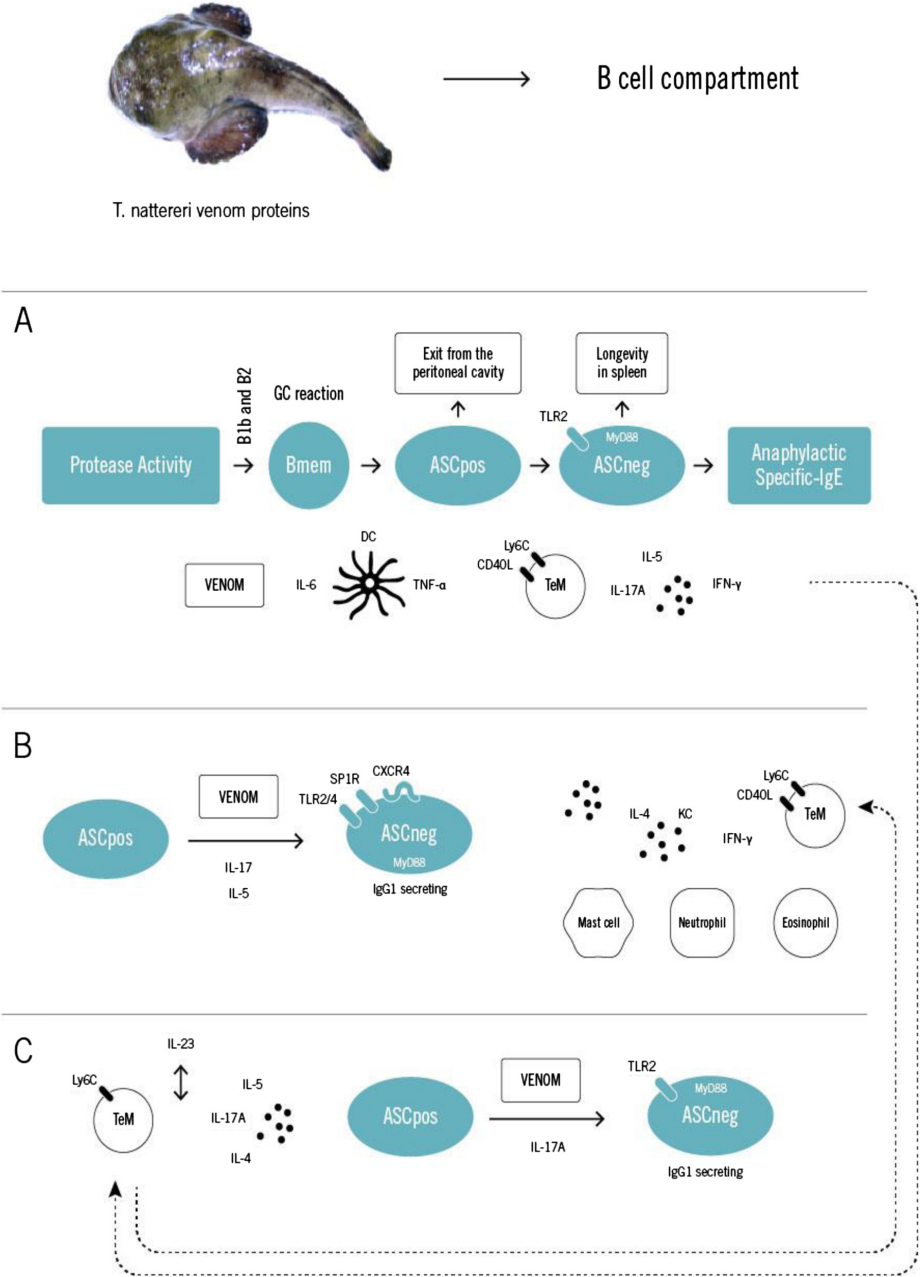
**Proposed model showing the effects of venom or isolated proteins on B cell compartment. (A)** ASC B220^neg^ are generated in GC of spleen by a hierarchical process of differentiation in which Bmem starts with high expression of B220 that reach intermediate levels and become ASC negative for this marker in a IL-17A-dependent manner. The longevity of ASC B220^neg^ in spleen is supported by TLR2 signaling. **(B)** IL-17A and IL-5 produced by innate cells that depend on TLR2/4 MyD88-signaling directly influences the differentiation and maintenance of ASC B220^neg^ producing specific-IgG1 on inflamed peritoneal cavity. The longevity of ASC B220^neg^ is supported by CXCR4 expression dependent on SP1R expression. **(C)** IL-5 and IL-23 maintain central memory T lymphocytes (TcM) in BM that migrate to secondary lymphoid organs where they could be activated and sustain antibody production by ASC.

In particular, memory response to venom induced a chronic expansion of B1a cells in bone marrow (BM), retention of venom proteins by splenic cells and maintenance of a Th2-mediated inflammation in the peritoneal cavity with infiltration of eosinophils, mast cells, neutrophils and IL-17A-producing effector memory T CD4 cells [29] (Figure 6). Then, we confirmed in an *in vitro* model the existence of a hierarchical process in which CD19-positive Bmem become CD138-positive IgG producing-ASC by a mechanism directly dependent on B-cell receptor (BCR) stimulation by venom, which could be potentiated by IL-17A [30]. Collectively, these studies shed new light on survival factors involved in the differentiation and maintenance of ASC in inflamed tissue and demonstrated that these cells require signals derived from antigen and IL-17A for the secretion of venom-specific antibodies for long periods of time.

Although the mechanisms leading to IgE-mediated Th2 response induced by proteases of allergens from various sources had been investigated, no information is available about the regulation of differentiation of Bmem or ASC by fish proteases [31-38]. Therefore, we examined the memory B cell development in different compartments upon natterins recall *in vivo*. We confirmed that natterins are dependent on proteolytic activity to lead the exit of Bmem and ASC B220^pos^ from the peritoneal cavity and from the BM, favoring the resting of ASC B220^neg^ in spleen. In addition, high-affinity specific IgG1 and IgE response dependent on the protease activity of natterins is produced by memory B cells and ASC generated in the GC after innate-like B1b or B2 activation [39].

The participation of TLR and MyD88 signals in the traffic of innate B cells and final differentiation of ASC in mice immunized with natterins were also investigated. Komegae *et al.*[40] demonstrated that TLR2-TLR4 derived signals (MyD88-dependent) modulate the emigration of innate-like B cells, Bmem and ASC B220^pos^ out of the peritoneal cavity sustaining the hierarchical differentiation into the spleen. Finally, we showed that the longevity of ASC B220^neg^ into inflamed peritoneal cavity induced by natterins is strong supported by up-regulated CXCR4 expression dependent on SP1R signals in B lymphocytes.

## Conclusion

The evolutionary pressure that selected venomous fish of Batrachoididae family created a distinct combination of toxic components with particular noxious characteristics. The toxic components of *T. nattereri* fish venom are distinguished by sensing-receptors of mammals immune system which control the induction of deleterious or beneficial responses. Nattectin acts as adjuvant inducing relevant cytokines and up-regulating the expression of co-stimulatory molecules in innate cells as antigens presenting cells that trigger a Th1 cell response with IgG1 specific-antibody production. Natterins act as tissue-damaging toxins promoting stasis and impairment of cell infiltration into inflamed tissue, with consequent delay of regeneration. Interestingly, we confirmed that the antagonist effect of natterins is mediated by the activation of serine/threonine phosphatases and by the key signaling PI3K molecule. Our findings of memory response reported herein show the increased capacity of *T. nattereri* venom proteins to modulate the memory B cell compartment favoring the development of long-lasting immunity. We suggest that a better understanding of this complexity of fish venom response will lead to more answers on how different antigenic epitopes activate the immune system and sustain the immune memory.

## Competing interests

The authors declare that they have no competing interests.

## Authors’ contributions

All authors contributed equally to the writing and revision of the article. All authors read and approved the final manuscript.
